# Variation in xenobiotic transport and metabolism genes, household chemical exposures, and risk of childhood acute lymphoblastic leukemia

**DOI:** 10.1007/s10552-012-9947-4

**Published:** 2012-06-07

**Authors:** Anand P. Chokkalingam, Catherine Metayer, Ghislaine A. Scelo, Jeffrey S. Chang, Kevin Y. Urayama, Melinda C. Aldrich, Neela Guha, Helen M. Hansen, Gary V. Dahl, Lisa F. Barcellos, John K. Wiencke, Joseph L. Wiemels, Patricia A. Buffler

**Affiliations:** 1 Division of Epidemiology, UC Berkeley School of Public Health, University of California Berkeley, 1995 University Ave, Ste 460, Berkeley, CA 94704 USA; 2 International Agency for Research on Cancer, Lyon, France; 3 Department of Epidemiology and Biostatistics, University of California San Francisco, San Francisco, CA USA; 4 National Institute of Cancer Research, National Health Research Institutes, Tainan, Taiwan; 5 Institute for Medicine and Public Health, Vanderbilt University, Nashville, TN USA; 6 Department of Neurological Surgery, University of California San Francisco, San Francisco, CA USA; 7 Lucile Packard Children’s Hospital, Stanford, CA USA

**Keywords:** Xenobiotic, Chemicals, California, Childhood cancer, Epidemiology

## Abstract

**Background:**

Recent studies suggest that environmental exposures to pesticides, tobacco, and other xenobiotic chemicals may increase risk of childhood acute lymphoblastic leukemia (ALL). We sought to evaluate the role of genes involved in xenobiotic transport and metabolism in childhood ALL risk, both alone and in conjunction with household chemical exposures previously found to be associated with childhood ALL risk.

**Methods:**

We conducted a population-based epidemiologic study of 377 cases and 448 controls in California, utilizing a haplotype-based approach to evaluate 42 xenobiotic transport and metabolism genes in conjunction with data on self-reported household chemical exposures.

**Results:**

We identified significant associations of childhood ALL risk with haplotypes of *ABCB1*, *ARNT*, *CYP2C8*, *CYP1A2*, *CYP1B1*, and *IDH1*. In addition, certain haplotypes showed significant joint effects with self-reported household chemical exposures on risk of childhood ALL. Specifically, elevated risks associated with use of paints in the home (ever) and indoor insecticides (pre-birth) were limited to subjects carrying specific haplotypes of *CYP2C8* and *ABCB1*, respectively.

**Conclusions:**

Our results provide support for a role of xenobiotic transport and metabolism pathways in risk of childhood ALL and indicate that genes in these pathways may modulate the risk of disease associated with use of common household chemicals. Additional studies are needed to confirm these findings and localize specific causal variants.

**Electronic supplementary material:**

The online version of this article (doi:10.1007/s10552-012-9947-4) contains supplementary material, which is available to authorized users.

## Introduction

Leukemia is the most common cancer among children under 15 years of age, accounting for 32 % of all childhood malignancies [1]. In California, Hispanics have the highest reported annual age-adjusted childhood leukemia incidence (56.2 per million), followed by non-Hispanic whites, Asian/Pacific Islanders, and non-Hispanic blacks (44.6, 40.0, and 29.1 per million, respectively) [2]. Although acute lymphoblastic leukemia (ALL) is the most common subtype of childhood leukemia, comprising ~80 % of total disease [1], it is much rarer than most cancers in adults and consequently more difficult to study epidemiologically. The etiology of ALL in children is believed to be distinct from that in adults, due largely to the clearer role for early life exposures. However, few risk factors have been conclusively established, including ionizing radiation, chemotherapeutic agents, and specific genetic abnormalities [3].

Recent studies from our group and others have found elevated childhood ALL risks associated with self-reported use of pesticides at home [4], household paint exposure [5], paternal smoking before conception [6, 7], and surrogate measures of exposure to motor vehicle exhaust [8–11]. In order to exert their effects, these potentially harmful xenobiotic (exogenous) chemicals must first gain entry into target cells and undergo cellular metabolic processes that alter their activity. Membrane transporters such as those encoded by the multiple drug resistance (*ABCB1*/*MDR1*) gene act as efflux pumps to expel compounds from the cell and are strategically expressed in regions of the body that act as epithelial barriers or perform excretory functions [12]. In addition, enzymes involved in phase I (bioactivation) and phase II (detoxification) metabolism maintain a critical balance of activation and inactivation of a wide range of chemical exposures of relevance to childhood ALL, including drugs, chemical carcinogens, insecticides, petroleum products, nitrosamines, polycyclic aromatic hydrocarbons, and other environmental pollutants [13].

In order to shed light on the role of genes involved in xenobiotic transport and metabolism, both alone and in conjunction with household chemical exposures, in childhood ALL risk, we utilized a haplotype-tagging approach to characterize genetic variation in a population-based study of 377 ALL case children and 448 control children in Northern and Central California. Furthermore, we examined whether effects of exposure to common household chemicals (including paints, solvents, pesticides, herbicides and tobacco smoke) previously linked to risk of childhood ALL or other childhood cancers were modified by these genetic variants.

## Subjects and methods

### Study population

The study was conducted among children participating in the Northern California Childhood Leukemia Study (NCCLS), an ongoing population-based case–control study conducted since 1995. The current study includes only those subjects for whom genotyping data were available. Enrollment and recruitment procedures have been described in detail previously [14]. Briefly, case children with incident childhood leukemia were ascertained via a rapid reporting system between the study office and participating hospitals. Control children identified from the state birth certificate records were individually matched to case children on date of birth, sex, maternal race, and child’s Hispanic ethnicity (having one or more parents reporting Hispanic ethnicity). Participation rates among eligible cases and controls were 87 and 86 %, respectively. Data on various potential risk factors were elicited from a parent (usually the mother) by trained interviewers using a structured questionnaire.

This study was reviewed and approved by institutional review committees at the University of California Berkeley, the California Department of Public Health (CDPH), and the participating hospitals. Written informed consent was obtained from all parent respondents.

### DNA specimens

Buccal cytobrushes collected from 95 % of participating children (cases and controls) enrolled between 1995 and 2002 were processed within 48 h of collection by heating in the presence of 0.5 N NaOH. Isolated DNA was later re-purified using an automated DNA extraction system (AutoGen, Holliston, MA) and then whole-genome amplified using GenomePlex reagents (Rubicon Genomics, Ann Arbor, MI). When buccal cytobrush DNA was inadequate (26.6 % of subjects), DNA was isolated from dried bloodspot specimens (DBSs) collected at birth and archived by the Genetic Disease Screening Program of the CDPH. DBS specimens were available for 91 % of California-born participants (100 % of controls and 90 % of cases). After extraction (QIAamp 96 DNA Blood Kit, QIAGEN, Germany), these DNA samples were whole-genome amplified using REPLI-g reagents (QIAGEN). All DNA samples were quantitated using human-specific Alu-PCR to confirm a minimum level of amplifiable human DNA [15]. When analyzed using highly multiplexed GoldenGate genotyping (Illumina, San Diego, CA), whole-genome-amplified buccal cell DNA yields genotypes that are highly concordant with those from genomic DNA from peripheral blood [16]. We genotyped DNA specimens from both buccal cells and DBS for 9 subjects; genotype concordance between paired samples was 98.9 %.

### Genotyping

Based on consensus review following review of the literature by our investigative team, we selected 42 genes coding xenobiotic transport and metabolism enzymes. Using HaploView software [17] in conjunction with reference single nucleotide polymorphism (SNP) data from the 30 Caucasian trios in the HapMap project (Release 19, Build 34, http://www.hapmap.org) and the 23 Hispanics in the SNP500Cancer project (http://www.snp500cancer.nci.nih.gov), we applied the method of Gabriel et al. [18] to select haplotype-tagging SNPs (htSNPs) that captured at least 80 % of diversity for common haplotypes (>5 % frequency) in either reference group. As Hispanics are a recently admixed ethnic group, and 42 % of our study population is Hispanic (at least one parent reporting Hispanic ethnicity), we placed special emphasis on capturing haplotype structures in Hispanics. To maximize capture of potential regulatory regions, we included in the SNP selection 10-kb stretches both up- and down-stream from the gene boundaries reported in the UCSC Genome Browser.

Genotyping of the selected SNPs was attempted in 385 ALL cases and 456 controls using a custom Illumina GoldenGate panel. We applied an Illumina GenCall quality threshold of 0.25, and SNP-wise and subject-wise call rate thresholds of ≥90 and ≥95 %, respectively. Genotypes for duplicate DNA specimens from 59 subjects showed 99.1 % concordance. Genotyped SNPs with an observed minor allele frequency <5 % (*n* = 13), or showing significant deviation from Hardy–Weinberg equilibrium (*p* < 0.01, *n* = 2), in both Hispanic controls and non-Hispanic controls were excluded. After applying these data quality thresholds, data for 250 SNPs in the 42 selected genes (Supplementary Table A) were available for 377 ALL cases and 448 controls.

In addition, we determined deletion status for *GSTM1* and *GSTT1* using polymerase chain reaction methods described previously [19].

### Household chemical exposure assessment

Details of data collection for self-reported household chemical use in early childhood have been published elsewhere [5, 20, 21]. Briefly, utilizing an in-home interview with a structured questionnaire, we asked parents whether paints, stains, or lacquers (collectively called “paints”); adhesives or petroleum products, such as paint thinner, spot remover, paint remover, glue, solvent, gasoline, kerosene, or lubricating oil (collectively called “solvents”); professional lawn service and the use of weed control products (collectively called “outdoor herbicides”); or professional pest control services, insect repellents, flea foggers, and products to control ants, flies, cockroaches, spiders, termites, and plant/tree insects (collectively called “indoor insecticides”); were ever used in the home during specific time windows from the 3 months prior to pregnancy through either the child’s 3rd birthday or diagnosis age (reference age among controls). For this analysis, we focused on time windows for which the chemicals showed significant main effects in our previous analyses [5, 20, 21]. Accordingly, for paints and solvents, we censored exposures at the time window preceding the reference date (e.g., from birth to 1 year if the case was diagnosed between 1 and 2 years of age). For outdoor herbicides and indoor insecticides, we limited to exposures before birth. We also ascertained whether there were any tobacco smokers in the house from birth through the child’s 3rd birthday. For the purpose of this analysis, all exposures were classified as “ever/never” during the specified time window. The subjects in the current study comprise a subset of subjects in our previous reports linking household chemicals to childhood leukemia risk. In the current study subset, we found the observed associations of these household chemicals with risk of childhood ALL were consistent with our previous reports: paint use (OR = 1.42, 95 % CI: 1.06–1.92), outdoor herbicides before birth (OR = 1.46, 95 % CI: 1.04–2.04), and indoor insecticides before birth (OR = 1.29, 95 % CI: 0.97–1.72). We previously found solvents to be associated with childhood acute myeloid leukemia, and household passive tobacco exposure after birth showed joint effects with paternal smoking on childhood ALL risk. We found no main effects of these two exposures in our study sample.

### Statistical analysis

Using a set of 80 ancestry informative markers [22], we have previously calculated individual estimates of Amerindian, African, and European genetic ancestry [23] using structured association methods [24, 25]. We found no evidence of major confounding by estimated genetic ancestry (>10 %) over and above adjustment for self-reported race and ethnicity [23]. Thus, we used stratification or adjustment for the self-reported factors in our analyses.

As a preliminary step prior to haplotype analysis, we tested for potential interactions of individual htSNPs with Hispanic ethnicity in disease risk on a gene-by-gene basis using unconditional logistic regression and the likelihood ratio test at the 0.05 significance level, after adjusting for age, sex, and child’s race.

For haplotype analysis, we used a haplotype sliding window approach for the SNPs in each gene, as implemented in the haplo.stats package for R [26]. This approach examines sub-haplotypes using the full set of SNP data, with differently sized “windows” of adjacent alleles. This is an effective means of combining multi-locus data for Hispanics and non-Hispanics alike, as it is agnostic to differences in haplotype structure, provided no individual SNPs for a given gene have a strong differential effect by Hispanic ethnicity. Thus, if none of the SNPs in a given gene showed significant interaction with ethnicity at *p* ≤ 0.05, data for both ethnicities were combined for haplotype analyses; otherwise, the haplotype analysis for that gene was conducted separately for Hispanics and non-Hispanics. We utilized GrASP, a graphical tool [27] to display and visualize sliding window results (Supplementary Figure 1). Using haplotype trend regression [28], we estimated the magnitude of effect associated with risk haplotypes from the windows with the smallest global *p* values, collapsing haplotypes with <5 % frequency among controls into a “rare haplotypes” category. We tested the significance of potential interactions between self-reported household chemical exposures and risk haplotypes using the likelihood ratio test, focusing on haplotypes with significant main effects among all subjects (i.e., both Hispanics and non-Hispanics combined) in order to maximize statistical power to detect interaction effects. We report both nominal and false discovery rate (FDR)-adjusted *p* values for the interaction analysis, adjusted for the total number of interactions examined [29]. Lastly, for haplotype-chemical interactions significant at *p*_FDR_ ≤ 0.05, we derived effect estimates for household chemicals by haplotype status, applying to each subject the haplotypes with the highest inferred probability.

## Results

Due to the matched case–control design of the NCCLS, the distribution of age, gender, race, and ethnicity was comparable between the 377 ALL cases and the 448 controls (Table 1). The 42 xenobiotic transport and metabolism pathway genes we examined are listed in Supplementary Table A. We found that htSNPs in eight genes (*ABCC1*, *CYP1A2*, *CYP1B1*, *CYP2B6*, *CYP3A5*, *GSS*, *IDH1*, and *UGT1A9*) showed significant heterogeneity of effect between Hispanics and non-Hispanics (*p* ≤ 0.05); further analyses for these genes were stratified by ethnicity.

**Table 1:** Characteristics of childhood acute lymphoblastic leukemia cases and controls, NCCLS

Variable	Cases	Controls
	*n* (%)	*n* (%)
Total	377 (100.0)	448 (100.0)
Sex		
Male	200 (53.1)	237 (52.9)
Female	177 (46.9)	211 (47.1)
Age at diagnosis or reference date		
Under 1 year	12 (3.2)	19 (4.2)
1–5 years	243 (64.5)	282 (62.9)
6–10 years	85 (22.5)	95 (21.2)
11–14 years	37 (9.8)	52 (11.6)
Child’s self-reported ethnicity		
Non-Hispanic	221 (58.6)	269 (60.0)
Hispanic	156 (41.4)	179 (40.0)
Child’s self-reported race		
White	214 (56.8)	255 (56.9)
Black	15 (4.0)	16 (3.6)
Native American	7 (1.9)	8 (1.8)
Asian/Pacific Islander	25 (6.6)	35 (7.8)
Mixed	110 (29.2)	133 (29.7)
Do not know	6 (1.6)	1 (0.2)

	Mean percent (SE)	Mean percent (SE)
Genetic ancestry^a^		
African	7.3 (14.7)	7.1 (14.5)
Amerindian	24.2 (23.7)	22.2 (22.8)
European	68.5 (27.3)	70.7 (26.9)

^a^Individual percent genetic ancestry estimated using 80 ancestry informative markers (AIMs) as described in Aldrich et al. Presented data are means of individual estimated percentages (and standard errors) of each ancestral population among cases and controls

Results for genes with significant (*p* ≤ 0.05) haplotype effects that persisted through increasingly larger windows are presented in Supplementary Figure 1. Haplotype trend regression results estimating the magnitudes of effect for haplotypes with the lowest multi-SNP p-value in sliding window analyses are shown in Table 2.

**Table 2:** Haplotype trend regression results: xenobiotic transport and metabolism genes in childhood acute lymphoblastic leukemia risk, NCCLS

Gene	SNPs	Ethnic group^a^	Haplotype^b^	Control frequency^c^	Case frequency^c^	OR (95 % CI)	*p* value	Global *p* value
*ABCB1*	*rs2520464*, *rs12334183*, *rs1202179*, *rs17327442*	All	Rare haplotypes	0.022	0.016	0.42 (0.08, 2.09)	0.289	0.019
			G–A–A–T	0.080	0.107	1.66 (0.83, 3.32)	0.153	
			G–A–G–T	0.136	0.096	**0.44 (0.23, 0.85)**	**0.015**	
			G–A–A–A	0.142	0.165	1.25 (0.70, 2.24)	0.454	
			G–G–G–T	0.168	0.144	0.68 (0.39, 1.20)	0.185	
			A–A–A–T	0.452	0.472	Ref		
*ARNT*	*rs2228099*, *rs4379678*	All	Rare haplotypes	<0.001	<0.001	–	0.011	0.003
			G–G	0.064	0.101	**4.93 (1.94, 12.53)**	**0.001**	
			C–A	0.410	0.389	1.09 (0.72, 1.66)	0.675	
			G–A	0.526	0.511	Ref		
*CYP2C8*	*rs1934954*, *rs7073968*, *rs7077618*, *rs10509681*	All	Rare haplotypes	0.026	0.026	1.29 (0.36, 4.62)	0.699	0.046
			G–G–T–G	0.059	0.092	**3.18 (1.45, 6.95)**	**0.004**	
			A–G–T–A	0.239	0.260	1.57 (0.96, 2.56)	0.071	
			A–A–T–A	0.258	0.261	1.34 (0.83, 2.15)	0.232	
			A–A–A–A	0.417	0.361	Ref		
*GCLC*	*rs553822*, *rs3799696*	All	Rare haplotypes	0.015	0.032	**17.47 (2.63, 116.1)**	**0.003**	0.012
			A–G	0.297	0.287	1.23 (0.75, 2.02)	0.408	
			G–G	0.327	0.349	1.42 (0.89, 2.27)	0.145	
			A–A	0.361	0.332	Ref		
*CYP1A2*	*rs11854147*, *rs11072508*	NH	Rare haplotypes	0.046	0.022	**0.14 (0.03, 0.76)**	**0.022**	0.001
			G–G	0.117	0.081	0.49 (0.20, 1.19)	0.116	
			A–G	0.315	0.414	**2.19 (1.28, 3.77)**	**0.005**	
			G–A	0.522	0.483	Ref		
*CYP1B1*	*rs162557*, *rs162556*	NH	A–A	0.081	0.040	**0.11 (0.02, 0.56)**	**0.007**	0.007
			A–G	0.140	0.160	1.71 (0.80, 3.68)	0.169	
			G–G	0.301	0.339	1.18 (0.62, 2.24)	0.612	
			G–A	0.478	0.461	Ref		
*CYP2B6*	*rs3760657*, *rs2054675*, *rs8100458*, *rs3745274*	NH	Rare haplotypes	0.009	0.028	**16.92 (1.54, 186.3)**	**0.021**	0.037
			G–A–G–C	0.086	0.094	1.18 (0.48, 2.94)	0.719	
			A–G–A–A	0.207	0.245	1.53 (0.81, 2.90)	0.193	
			A–A–G–C	0.256	0.211	0.70 (0.36, 1.36)	0.292	
			A–A–A–C	0.442	0.423	Ref		
*IDH1*	*rs1992739*, *rs4290589*	Hisp	C–G	0.054	0.048	0.55 (0.11, 2.81)	0.474	0.008
			C–C	0.058	0.127	**6.12 (1.75, 21.36)**	**0.005**	
			G–G	0.144	0.103	0.61 (0.22, 1.73)	0.355	
			G–C	0.743	0.722	Ref		

^a^ *NH* non-Hispanics, *Hisp* Hispanics, *All* non-Hispanics and Hispanics combined

^b^ “Rare haplotypes” refers to combined group of individual haplotypes with <5 % frequency among controls

^c^377 childhood ALL cases, 448 controls

Among all subjects, *ABCB1*, *ARNT*, *CYP2C8*, and *GCLC* showed significant associations that persisted through progressively larger SNP windows (Table 2). In *ABCB1*, haplotype G–A–G–T was associated with a significantly reduced risk (OR = 0.44, *p* = 0.015). Haplotypes G–G of *ARNT* and G–G–T–G of *CYP2C8* were significantly associated with increased risks of childhood ALL (OR = 4.93 and *p* = 0.001, OR = 3.18 and *p* = 0.004, respectively). The observed significant global haplotype association of *GCLC* was attributed to a rare haplotype; no further analysis was performed for this gene.

Among non-Hispanics, *CYP1A2* and *CYP1B1* showed significant haplotype associations. Haplotype A–G of *CYP1A2* was significantly associated with an increased risk (OR = 2.19, *p* = 0.005), while haplotype A–A of *CYP1B1* was significantly associated with a decreased risk (OR = 0.11, *p* = 0.007). The observed significant global haplotype association of *CYP2B6* was attributed to rare haplotypes. Among Hispanics, the two SNPs in *IDH1* showed a haplotype association stronger than either SNP individually (global *p* = 0.008), and the C–C haplotype was significantly associated with an increased risk of ALL (OR = 6.12, *p* = 0.005).

Two of the most commonly studied xenobiotic metabolism genes to date in childhood ALL are the glutathione S transferase genes *GSTM1* and *GSTT1*, whose principal variants are deletions [30]. The *GSTM1* deletion showed significantly different effects by Hispanic ethnicity (*p* < 0.001): among Hispanics the deletion was associated with elevated risk (OR = 1.85, 95 % CI 1.19–2.88, *p* = 0.007) while among non-Hispanics, the association was in the opposite direction (OR = 0.62, 95 % CI 0.43–0.89, *p* = 0.010). In addition, there was no evidence of association for the *GSTT1* deletion (*p* = 0.526).

Finally, we examined interactions between household chemical exposures of interest and xenobiotic gene variants. For this analysis, we focused on haplotypes with at least 5 % frequency among controls and showed nominally significant main effects (global *p* ≤ 0.05) among Hispanics and non-Hispanics combined. We found significant interactions between *CYP2C8* haplotype G–G–T–G and self-reported use of paints after birth (*p*_FDR_ = 0.016), and *ABCB1* haplotype G–A–G–T and self-reported use of indoor insecticides before birth (*p*_FDR_ = 0.035) (Table 3). As shown in Table 4, our analysis indicates that the risks of childhood ALL associated with use of paints and indoor insecticides vary by presence or absence of these haplotypes. The increased risks associated with paint use appears confined to those with *CYP2C8* haplotype G–G–T–G (OR = 1.67, 95 % CI 1.21–2.30), while in the small subgroup without the G–G–T–G haplotype (5.9 % among controls), paint use appears to be associated with a non-significant reduced risk (OR = 0.45, 95 % CI = 0.20–1.02). Similarly, the increased risk associated with use of indoor insecticides appears to be limited to the small population with the *ABCB1* G–A–G–T haplotype (13.6 % among controls, OR for indoor insecticides = 3.03, 95 % CI = 1.59–5.78), while among those without the G–A–G–T haplotype, the risk associated with indoor insecticide use was null (OR = 1.02, 95 % CI = 0.74–1.41).

**Table 3:** Interactions of household chemical exposures with xenobiotic metabolism and transport genes on childhood acute lymphoblastic leukemia risk ^a^

Gene	Haplotype	Main effect *p*_Nominal_	Interaction with:									
			Paint use, ever		Solvent use, ever		Outdoor herbicide use, pre-birth		Indoor insecticide use, pre-birth		Any smokers in the home after birth	
			*p*_Nominal_	*p*_FDR_^b^	*p*_Nominal_	*p*_FDR_^b^	*p*_Nominal_	*p*_FDR_^b^	*p*_Nominal_	*p*_FDR_^b^	*p*_Nominal_	*p*_FDR_^b^
*CYP2C8*	Hap G–G–T–G	0.014	**0.001**	**0.016**	0.022	0.082	0.253	0.474	0.013	0.067	0.300	0.501
*ABCB1*	Hap G–A–G–T	0.013	0.850	0.931	0.587	0.834	0.869	0.931	**0.005**	**0.035**	0.612	0.834
*ARNT*	Hap G–G	0.009	0.699	0.874	0.028	0.083	0.996	0.996	0.068	0.146	0.042	0.105

^a^Analyses conducted for haplotypes with significant main effects (global *p* ≤ 0.05) among all ethnicities combined

^b^FDR adjusted for total number of interaction tests conducted (*n* = 15)

**Table 4:** Chemical by haplotype interaction analysis: effect sizes for childhood ALL risk

Haplotype	Exposure	*n* Cases	*n* Controls	Risk of childhood ALL	
				OR (95 % CI)	Wald *p*
CYP2C8 G–G–T–G	Paint in house, ever				
N	N	133	202	1.00 (ref)	
N	Y	178	195	1.67 (1.21–2.30)	**0.0020**
Y	N	29	13	1.00 (ref)	
Y	Y	37	38	0.45 (0.20–1.02)	0.0571
ABCB1 G–A–G–T	Indoor insecticide, pre-birth				
N	N	136	146	1.00 (ref)	
N	Y	174	186	1.02 (0.74–1.41)	0.8976
Y	N	21	65	1.00 (ref)	
Y	Y	46	51	3.03 (1.59–5.78)	**0.0008**

## Discussion

In this population-based case–control study, we examined the risk of childhood ALL associated with several genes within the xenobiotic transport and metabolism pathways, utilizing a haplotype-tagging approach to maximize capture of genetic variation. We identified haplotypes of several genes that were significantly associated with childhood ALL, including *ABCB1*, *ARNT*, *CYP2C8*, *CYP1A2*, *CYP1B1,* and *IDH1*. In addition, we observed significant interactions of identified risk haplotypes with a number of self-reported household chemical exposures, including use of paints and indoor insecticides. Although confirmation is required, our findings provide evidence that genes involved in the xenobiotic transport and metabolism pathway may play a role in mediating risk of childhood ALL, and that the childhood ALL risks associated with various household chemical exposures may be modified by these variation in these genes.

A haplotype of *ABCB1*, which encodes a membrane transporter of lipophilic compounds, was significantly associated with childhood ALL risk and showed significant interaction with indoor insecticides, mirroring an earlier finding utilizing different genetic variants in the same gene [31]. Our results indicate that the increased risk associated with use of indoor insecticides before birth is limited to subjects carrying the G–A–G–T haplotype. The SNPs in this risk haplotype are 21 kb from the nearest of the 3′ SNPs examined in our previous analysis, in which no significant haplotype main effect was observed [31]. This is in agreement with the current analysis, which also shows no main effect of haplotypes at the 3′ end of the gene.

We also found significant childhood ALL associations with haplotypes in three genes in the CYP gene family: *CYP2C8*, *CYP1A2*, and *CYP1B1*. The *CYP2C8* gene product is involved in metabolism of numerous drugs and other compounds [32]. In addition to a significant association with childhood ALL, the risk haplotype for *CYP2C8* showed significant interaction with self-reported household paint use, with the increased risk associated with paint use being limited largely to those without the *CYP2C8* G–G–T–G haplotype. For the common haplotype in *CYP1A2* (31.5 % frequency among controls), we found an elevated risk of childhood ALL. The SNPs composing this haplotype are outside the *CYP1A2* coding region, the nearest (rs11854147) being 5.4 kb from the 3′ end. The *CYP1A2* gene product metabolizes polycyclic aromatic hydrocarbons (PAHs, found in tobacco smoke and vehicle exhaust); in utero exposures to PAHs have been linked to chromosomal aberrations [33]. *CYP1B1*, for which we observed a significant haplotype association with childhood ALL risk, is also involved in metabolism of PAHs, as well as steroids [34].

The *ARNT* gene product is a key transporter of PAHs and other compounds, and a transcription inducer of xenobiotic metabolism genes including *CYP1A1* and *CYP1A2* [35] that metabolize PAHs. We identified a risk haplotype for *ARNT* that showed a markedly higher risk of childhood ALL. We also observed a strong haplotype association for *IDH1*; a somatic mutation in *IDH1* has been linked to survival in adult glioblastoma and AML [36, 37]. The two SNPs we examined are downstream from and in strong LD with SNPs in the *IDH1* coding region.

In gauging these results, consideration must be given to several factors. First, despite this study’s relatively large sample size compared to those of most previous candidate gene studies, the presence of genetic heterogeneity due to the ethnic and racial diversity of the California population may have influenced our ability to detect associations. Our SNP selection strategy included elements designed to maximize capture of genetic variation in Hispanics. We examined Hispanics separately from non-Hispanics where there was significant heterogeneity in between-group effects of individual SNPs. Although this approach may have limited our ability to detect associations in the population as a whole, we believe it was necessary given that genetic susceptibility may be different in Hispanics versus non-Hispanics due to the Hispanic population’s relatively recent genetic admixture [22]. Results that differ between Hispanics and non-Hispanics may be due to differences in allele frequency and/or haplotype structure or may reflect underlying differences in exposures that modulate the effects of genes. Regardless, if the results are not spurious, they represent potential risk loci, and we present them in either or both ethnic groups for replication and further followup. Whereas the entire study population yielded adequate power to detect modest effect sizes (81 % power for OR_log additive_ = 1.40, minor allele frequency = 20 %), power was lower among Hispanics and non-Hispanics separately (44 and 59 %, respectively). In addition, the limited size of racial/ethnic sub-populations within the non-Hispanic group precluded further stratification of this group; as such, genetic heterogeneity among non-Hispanics might have obscured results. However, we found no evidence of strong confounding due to estimated genetic ancestry [23], minimizing concerns about the impact of population stratification on the results.

Two large genome-wide association studies on childhood ALL have been published to date (with 907 cases and 2,398 adult and child controls, and 317 cases and 17,958 adult controls, respectively) [38, 39]. Although these studies have identified a number of novel loci, no significant associations were observed for genes in the pathways we studied here. Null findings for these genes in the genome-wide studies may be due to stringent multiple testing adjustment (at the *p* ≤ 1 × 10^−7^ level) to account for the large number of individual variants under study. In contrast to the agnostic approach to discovery used in genome-wide studies, our study focused on relatively few genes representing key elements of the xenobiotic transport and metabolism pathways. We concede that results of our study may be due to chance and therefore must be replicated. However, the haplotype-tagging approach we adopted maximizes capture of total variation within each candidate gene and the haplotype analysis increases statistical power to detect associations over analyses of individual variants. Furthermore, although the haplotype-tagging approach does not pinpoint potential causal SNPs, it does localize risk-associated regions for further investigation such as fine-mapping.

In this study, we examined potential interactions of xenobiotic transport and metabolism genes with self-reported household chemical exposures early in childhood in the modulation of childhood ALL risk, focusing on haplotype findings observed for both ethnicities (Hispanics and non-Hispanics) combined, as the sizes of the individual ethnic groups were considered too small to permit adequately powered examinations of gene–environment interactions. Our observation that the increased risk associated with paint and indoor insecticide use [5, 20] was limited to specific subgroups defined by haplotypes of specific genes is suggestive that these genes work in concert with chemical use to modulate risk. Although we focused on a limited number of biologically plausible interactions according to a rigorous a priori analysis plan, we acknowledge that this analysis might be considered exploratory. As such we report only those interactions that were significant after accounting for multiple hypothesis testing. We recognize that our total sample size (377 cases, 448 controls) may be insufficient to observe modest interaction effects with adequate statistical power. Furthermore, although the environmental chemical exposures we examined have been previously associated with childhood leukemia risk [5, 21], these measures are derived from maternal self-reports, which are prone to reporting errors and recall bias in that mothers of cases may recall exposures differently than mothers of controls. Since this study was population-based with participation rates of 86–87 % and biospecimen collection rates >95 % for interviewed subjects, it is unlikely the results presented here are driven by bias in selection or participation. Further studies with improved measures of chemical exposure are needed to confirm the interactions observed.

In summary, we set out to investigate the role of genes in the xenobiotic transport and metabolism pathway in risk of childhood ALL in greater depth and with larger sample size than previous candidate gene studies. We also sought to examine the putative joint effects of these genes with environmental chemical exposures for which we have observed significant main effects. Our results provide evidence that elements of the xenobiotic transport and metabolism pathway may be associated with childhood ALL, and that some of these elements interact with chemical exposures to modulate risk. This study does not address the potential effects of maternal genes, which may *influence* in utero susceptibility to chemical exposures. The associations and interactions identified should be considered targets for further study in additional studies, with larger sample sizes, high quality environmental exposure data, maternal genes, and finer coverage of SNPs in the identified associated regions.

## Electronic supplementary material

Below is the link to the electronic supplementary material.

Supplementary material 1 (PDF 86 kb)

Supplementary material 2 (PDF 54 kb)

## Abbreviations

ALL: Acute lymphoblastic leukemia
CDPH: California Department of Public Health
CI: Confidence interval
DNA: Deoxyribonucleic acid
DBS: Dried bloodspot specimen
FDR: False discovery rate
htSNP: Haplotype-tagging SNP
NCCLS: Northern California Childhood Leukemia Study
OR: Odds ratio
SNP: Single nucleotide polymorphism
