# Supplementary material for: Variation in xenobiotic transport and metabolism genes, household chemical exposures, and risk of childhood acute lymphoblastic leukemia
Source: Cancer Causes Control. 2012 Jun 7;23(8):1367–75. doi: 10.1007/s10552-012-9947-4 (PMC3390694; doi:10.1007/s10552-012-9947-4)
Supplement: Supplementary file 1 — Supplementary material 1 (PDF 86 kb) [file 10552_2012_9947_MOESM1_ESM.pdf]

Supplementary Figure 1. Haplotype sliding window analyses for xenobiotic transport and metabolism genes with significant haplotype results (p≤0.05)

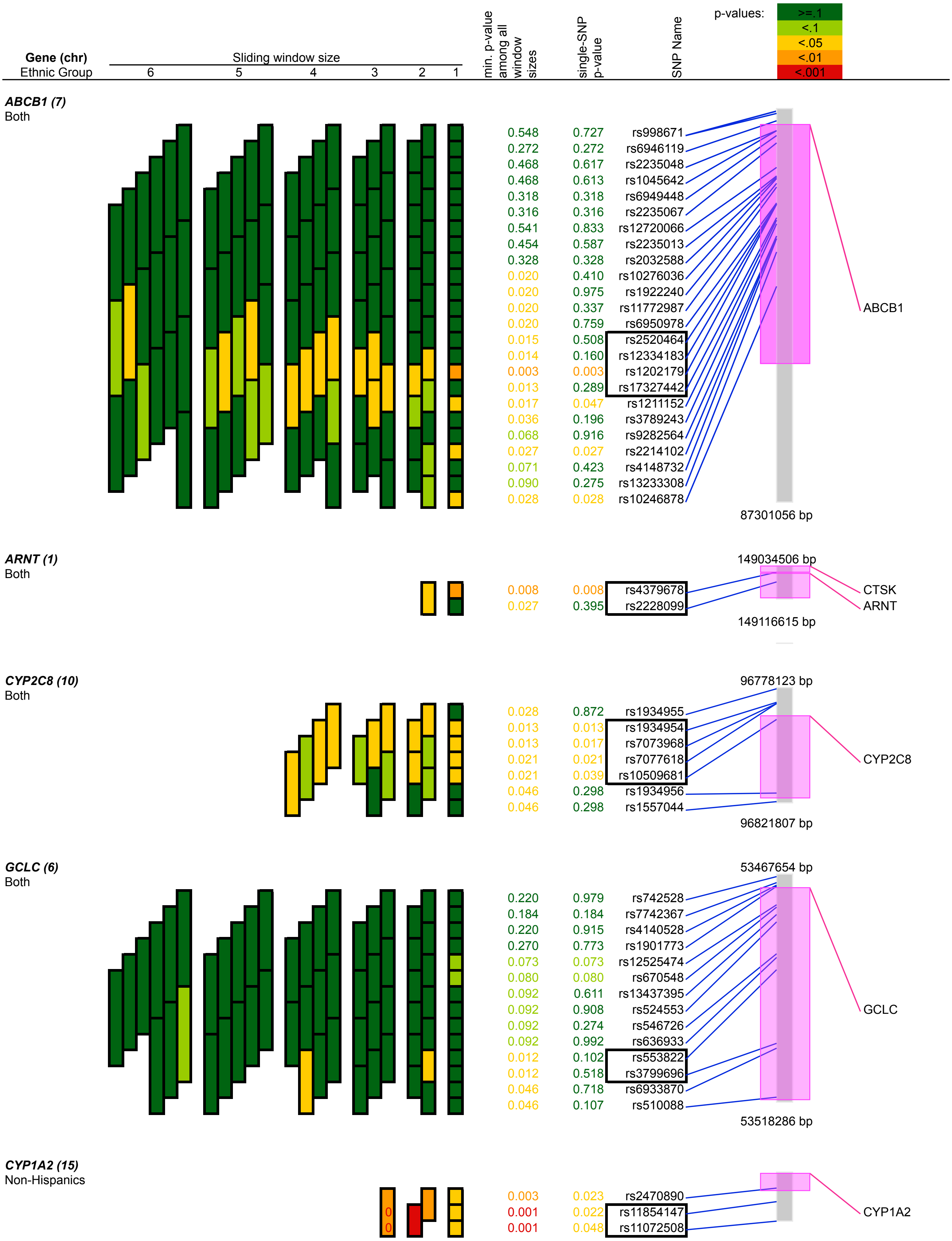

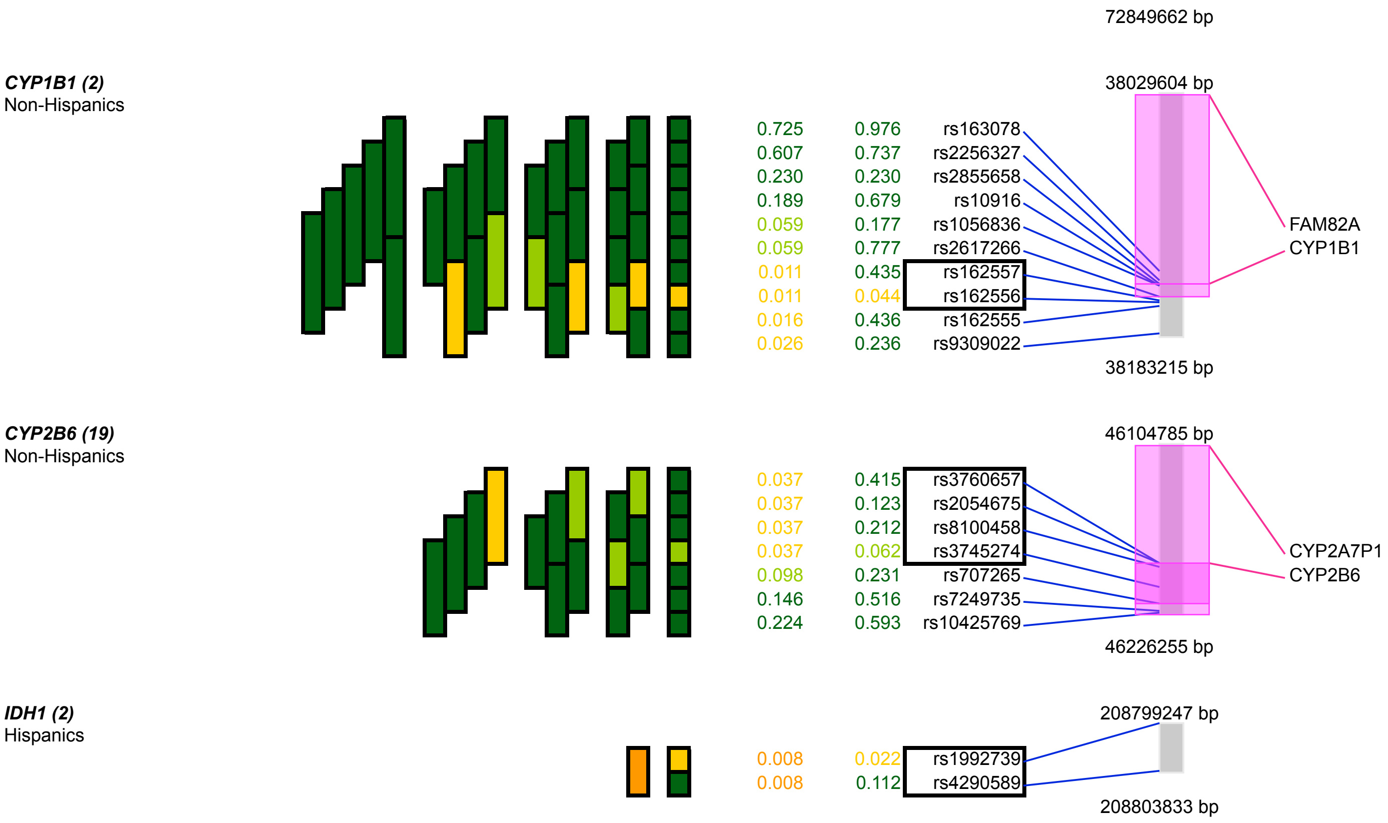

72849662 bp
CYP1B1 (2)
Non-Hispanics
38029604 bp
0.725 0.976 rs163078
0.607 0.737 rs2256327
0.230 0.230 rs2855658
0.189 0.679 rs10916
0.059 0.177 rs1056836
0.059 0.777 rs2617266
0.011 0.435 rs162557
0.011 0.044 rs162556
0.016 0.436 rs162555
0.026 0.236 rs9309022
FAM82A
CYP1B1
38183215 bp
CYP2B6 (19)
Non-Hispanics
46104785 bp
0.037 0.415 rs3760657
0.037 0.123 rs2054675
0.037 0.212 rs8100458
0.037 0.062 rs3745274
0.098 0.231 rs707265
0.146 0.516 rs7249735
0.224 0.593 rs10425769
CYP2A7P1
CYP2B6
46226255 bp
IDH1 (2)
Hispanics
208799247 bp
0.008 0.022 rs1992739
0.008 0.112 rs4290589
208803833 bp
