# Supplementary material for: Variation in xenobiotic transport and metabolism genes, household chemical exposures, and risk of childhood acute lymphoblastic leukemia
Source: Cancer Causes Control. 2012 Jun 7;23(8):1367–75. doi: 10.1007/s10552-012-9947-4 (PMC3390694; doi:10.1007/s10552-012-9947-4)
Supplement: Supplementary file 2 — Supplementary material 2 (PDF 54 kb) [file 10552_2012_9947_MOESM2_ESM.pdf]

Supplementary Table A. Xenobiotic transport and metabolism genes and single nucleotide polymorphisms (SNPs) typed

Gene symbol	Gene name	Chromosome	N total haplotype tagging SNPs typed
ABCB1 (MDR1)	ATP-binding cassette, sub-family B (MDR/TAP), member 1	07	24
ABCC1 (MRP1)	ATP-binding cassette, sub-family C (CFTR/MRP), member 1	16	39
ABCC2 (MRP2)	ATP-binding cassette, sub-family C (CFTR/MRP), member 2	10	8
AHR	aryl hydrocarbon receptor	07	1
ARNT	aryl hydrocarbon receptor nuclear translocator	01	2
COMT	catechol-O-methyltransferase	22	5
CYP1A1	cytochrome P450, family 1, subfamily A, polypeptide 1	15	6
CYP1A2	cytochrome P450, family 1, subfamily A, polypeptide 2	15	3
CYP1B1	cytochrome P450, family 1, subfamily B, polypeptide 1	02	10
CYP2A6	cytochrome P450, family 2, subfamily A, polypeptide 6	19	1
CYP2B6	cytochrome P450, family 2, subfamily B, polypeptide 6	19	7
CYP2C19	cytochrome P450, family 2, subfamily C, polypeptide 19	10	4
CYP2C8	cytochrome P450, family 2, subfamily C, polypeptide 8	10	7
CYP2D6	cytochrome P450, family 2, subfamily D, polypeptide 6	22	3
CYP2E1	cytochrome P450, family 2, subfamily E, polypeptide 1	10	5
CYP3A4	cytochrome P450, family 3, subfamily A, polypeptide 4	07	2
CYP3A5	cytochrome P450, family 3, subfamily A, polypeptide 5	07	3
EPHX1	epoxide hydrolase 1, microsomal (xenobiotic)	01	4
EPHX2	epoxide hydrolase 2, cytoplasmic	08	7
FMO3	flavin containing monooxygenase 3	01	6
GCLC	glutamate-cysteine ligase, catalytic subunit	06	14
GGT1	gamma-glutamyltransferase 1	22	1
GPX6	glutathione peroxidase 6 (olfactory)	06	4
GSR	glutathione reductase	08	5
GSS	glutathione synthetase	20	5
GSTA1	glutathione S-transferase A1	06	2
GSTO2	glutathione S-transferase omega 2	10	3
GSTP1	glutathione S-transferase pi	11	1
IDH1	isocitrate dehydrogenase 1 (NADP+), soluble	02	2
MPO	myeloperoxidase	17	3
NAT1	N-acetyltransferase 1 (arylamine N-acetyltransferase)	08	6
NAT2	N-acetyltransferase 2 (arylamine N-acetyltransferase)	08	11
NQO1	NAD(P)H dehydrogenase, quinone 1	16	4
PON1	paraoxonase 1	07	11
PTGS1 (COX1)	prostaglandin-endoperoxide synthase 1 (prostaglandin G/H synthase and cyclooxygenase 1)	09	2
PTGS2 (COX2)	prostaglandin-endoperoxide synthase 2 (prostaglandin G/H synthase and cyclooxygenase 2)	01	4
SULT1A1	sulfotransferase family, cytosolic, 1A, phenol-preferring, member 1	16	2
TPMT	thiopurine S-methyltransferase	06	4
UGT1A1	UDP glucuronosyltransferase 1 family, polypeptide A1	02	6
UGT1A7	UDP glucuronosyltransferase 1 family, polypeptide A7	02	5
UGT1A9	UDP glucuronosyltransferase 1 family, polypeptide A9	02	4
UGT2B7	UDP glucuronosyltransferase 2 family, polypeptide B7	04	4
